# The Use of a DCIA Free Flap with Internal and External Oblique Abdominal Muscle in Compound Oral Cavity Defects: A Pilot Study

**DOI:** 10.3390/jcm14186405

**Published:** 2025-09-11

**Authors:** Katarzyna Iwulska, Marcin Czajka, Drążek Jacek, Dubis Przemysław, Mariusz Szuta

**Affiliations:** 1 Department of Maxillofacial Surgery, Rydygier Hospital, os. Złotej Jesieni 1, 31-826 Kraków, Poland; mar_cz@poczta.onet.pl (M.C.); jacekdrazek777@gmail.com (D.J.); dubis.przemek@gmail.com (D.P.); 2 Chair of Oral Surgery, Medical College, Jagiellonian University, Montelupich 4, 31-155 Kraków, Poland; m.szuta@wp.pl

**Keywords:** DCIA, oblique muscle, maxillofacial

## Abstract

**Background/Objectives**: The deep circumflex iliac artery (DCIA) free flap with internal abdominal oblique muscle (IAOM) is a well-known method of reconstruction used in cases of oral cavity neoplasms. Because the IAOM can be insufficient for extensive defects after removal of advanced carcinomas of the tongue, floor of the mouth, or gingiva, the additional preparation of a perforator-supported external abdominal oblique (EAOM) muscle flap can be useful. The aim of this study was to introduce the use of a DCIA flap with an IAOM and EAOM island in the reconstruction of oral cavity compound defects. **Methods**: A retrospective analysis was performed involving eight patients who underwent reconstruction using a DCIA free flap with IAOM and perforator-supported EAOM island. Patients underwent the operation between June 2021 and February 2025 in the Department of Maxillofacial Surgery of the Rydygier Hospital in Kraków, Poland. **Results**: A group of eight patients underwent an operation due to squamous cell carcinoma of the oral cavity. The most common primary subsite of disease was the floor of the mouth (*n* = 4, 50%), followed by the lower gingiva (*n* = 2, 25%) and retromolar area (*n* = 2, 25%). All patients required resection involving part of the mandible, the floor of the mouth, and part of the tongue simultaneously with reconstruction using a DCIA free flap with IAOM and perforator-supported EAOM island. Osteotomies were performed in two flaps (one single osteotomy, one double osteotomy). Reconstruction was successfully performed in seven out of eight patients (overall success rate 88%). **Conclusions**: The DCIA free flap with IAOM and perforator-supported EAOM flap is a reliable method for compound soft tissue and bone defects in maxillofacial reconstruction. The use of IAOM and EAOM can be helpful in cases of three-dimensional soft tissue defects of the lower gingiva, the floor of the mouth, and the tongue. The lower gingiva and floor of the mouth can be reconstructed with IAOM, while the more mobile part of the tongue can be reconstructed with a perforator-supported EAOM island.

## 1. Introduction

With the advancement of technology and the development of microsurgical techniques, the field of maxillofacial surgery is rapidly changing. Currently, microsurgical reconstruction, which enables subsequent prosthetic dental rehabilitation, is becoming a standard procedure even in patients who have undergone extensive resections in the lower part of the face [1]. Large defects after the excision of malignant tumors are often multidimensional and require bone and soft tissue reconstruction in various planes. Invasive squamous cell carcinomas of the oral cavity at an advanced local stage (T3, T4) often simultaneously infiltrate the floor of the mouth, lower gingiva, and tongue. Various flap modifications are introduced to improve surgical methods in order to restore the shape and function of the resected areas, and at the same time decrease operation time or limit donor site morbidity [1,2,3]. The deep circumflex iliac artery flap (DCIA) was first reported by Taylor and has undergone a gradual evolution to provide a more functional oromandibular reconstruction, and from that time, various modifications have occurred [2,3,4,5]. Ramasastry et al. [5] investigated the myo-osseous variant of the DCIA, while Urken at al. [6] described the osteomyocutaneous DCIA. Zhang et al. [7] described the iliac bone flap with the perforator-supported external oblique abdominal muscle (EAOM) island technique for buried flap monitoring. The aim of this study was to introduce the use of a DCIA flap with internal oblique abdominal muscle IAOM and a perforator-supported EAOM island in the reconstruction of oral cavity compound defects. However, to the best of our knowledge, we have not found similar reports concerning the usage of EAOM in oral cavity reconstructions in English language publications.

## 2. Materials and Methods

Our comprehensive retrospective study encompassed medical chart review of patients who underwent operations between June 2021 and February 2025 in the Department of Maxillofacial Surgery of the Rydygier Hospital in Kraków, Poland. The investigated study group consisted of 8 patients undergoing segmental jaw resection and primary reconstruction using a DCIA free flap with IAOM and perforator-supported EAOM island. In one patient, an additional perforator-supported skin island was raised. Written informed consent was obtained from all patients before surgery. The study was approved by the Ethics Committee (Nr 105/KBL/OIL/2024) and adhered to the principles outlined in the Declaration of Helsinki. All patients provided written informed consent.

### 2.1. Characteristics of the Study Group

Inclusion criteria were as follows: advanced squamous cell carcinoma involving the mandible/lower gingiva, floor of the mouth, and tongue in patients who had undergone resection and reconstruction using a modified DCIA free flap with IAOM and EAOM perforator-supported island, complete clinical and radiological documentation, and a minimum follow-up of at least 3 months. Patients with a defect of the mandible, lower gum, and floor of the mouth and an adjacent soft tissue defect which required reconstruction with an additional mobile flap placed in a different plane (for example part of the tongue, the throat, or the area of the maxillary tuberosity) were qualified for reconstruction using a DCIA flap with IAOM and EAOM.

Pathological staging was performed according to the 8th edition of the International Union Against Cancer Control/American Joint Committee on Cancer Staging System TNM Classification. All patients underwent tumor resection and neck dissection according to National Comprehensive Cancer Network (NCCN) guidelines simultaneously with consecutive reconstruction. Post-operative adjuvant radiation therapy (RT) or concurrent chemo-radiation was applied for indicated patients according to NCCN guidelines. The mean follow-up duration was 15 months (range 4 to 34 months).

### 2.2. Surgical Methods

All patients underwent a computed-tomography (CT) scan of the head, neck, and pelvic region before surgery. Patient-specific cutting guides were prepared in order to facilitate tumor resection and osseous DCIA flap harvesting. The cutting guides were designed using Blender (version 2.78), a free software. Pre-operatively, the perforator to the external oblique muscle was localized using ultrasound Doppler (Minidop ES-100VX, Hadeco, Inc., Kawasaki, Japan) examination.

At the beginning of the operation, the important anatomical points were marked, such as the DCIA, the anterior superior iliac spine (ASIS), and the course of the curvature of the iliac crest. Incision of the skin and subcutaneous tissue was made between the femoral artery and the ASIS, and along the iliac crest. Incision length along the iliac crest depended on the location of the planned osteotomy lines and the size of the harvested bone fragment. After exposing the inguinal ligament and EAOM, incision of the fascia and EAOM was performed. The EAOM was dissected approximately 4–5 cm above the iliac crest posterolaterally and then the perforator vessel between the IAOM and EAOM was exposed, in most cases located approximately 4–6 cm posterior to the ASIS and 1–3 cm above the iliac crest. After identifying the perforator, careful dissection was carried out to expose the vessel between the abdominal muscles. Then, the ellipse-shaped EAOM island was harvested with dimensions of approximately 3–5 cm in width. The EAOM perforator island was temporarily sutured to the IAOM surface so as not to damage the island-nourishing vessels. Next, further dissection and harvesting of the myo-osseous iliac crest flap with IAOM was continued, employing the technique described by Urken [6]. Tumor resection and parallel elevation of the flap were accomplished by a two-team approach. For donor site closure, a synthetic mesh was placed between the transversus abdominus muscle layers and the external oblique layers and attached to the bone and margins of the internal oblique muscle. In all cases, the EAOM was closed by primary suturing. Flap-raising in all patients was performed by the same team of two experienced surgeons.

## 3. Results

A total of eight patients were included in the study. The median age was 60 years (range 39 to 73 years). In our group of patients, four were female (50%) and four male (50%). Patient characteristics are summarized in Table 1. The most common primary subsite of disease was the floor of the mouth (*n* = 4, 50%), followed by the lower gingiva (*n* = 2, 25%) and the retromolar area (*n* = 2, 25%). All patients required resection involving part of the mandible, the floor of the mouth, and part of the tongue. According to Brown’s (2016) classification [8], mandibular defects included class I (*n* = 1, 12.5%), class II (*n* = 2, 25%), and class III (*n* = 5, 62.5%). Two patients required partial resection of the middle throat and tonsils, while one patient required additional resection of the maxillary tuberosity.

**Figure 1:**
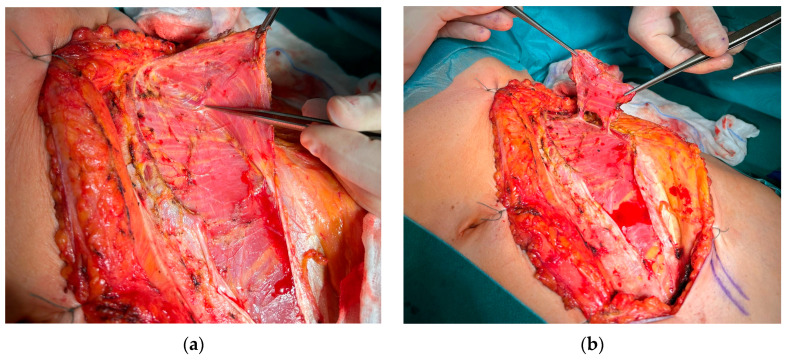
Preparation of a DCIA flap with IAOM and a perforator-supported EAOM island. (**a**) Pointed perforator to EAOM. (**b**) IAOM and perforator-supported EAOM island.

**Figure 2:**
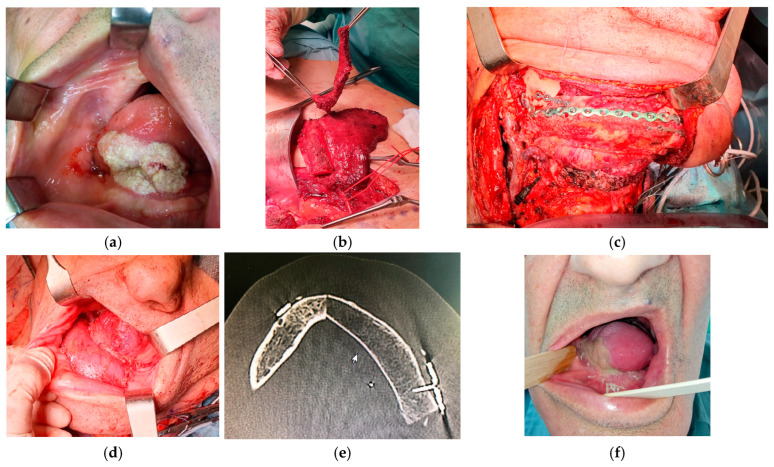
Reconstruction using a DCIA free flap with IAOM and a perforator-supported EAOM island. (**a**) Squamous cell carcinoma of the tongue and floor of the mouth. (**b**) Elevated perforator-supported EAOM island. (**c**) Intraoperative view. (**d**) The patient immediately post-operation: reconstruction of the lower gingiva and floor of the mouth with IAOM and tongue with a perforator-supported EAOM island. (**e**) Postoperative computed-tomography scan. (**f**) The patient’s appearance 1 month after surgery.

**Figure 3:**
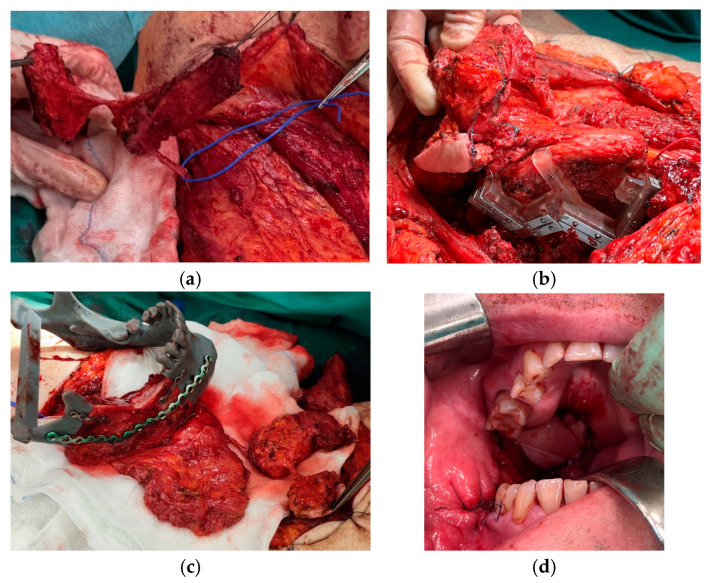
Reconstruction using a DCIA free flap with IAOM, a perforator-supported EAOM island and skin island. (**a**) Pointed perforator to EAOM and skin island. (**b**) DCIA flap with IAOM, a perforator-supported EAOM island, and a skin island with a cutting-guide. (**c**) Prepared DCIA flap with IAOM, a perforator-supported EAOM island, and a skin island. (**d**) The patient immediately post-operation: an EAOM island was used to close the middle throat defect and the skin island was used to close the maxillary tuberosity defect.

All patients underwent tumor resection and neck dissection simultaneously with reconstruction using a DCIA free flap with IAOM and perforator-supported EAOM island. In one patient, an additional skin flap perforator was harvested. In seven patients, a single dominant perforator was found, while in one patient, two perforators were found [Figure 1]. Osteotomies were performed in two flaps (one single osteotomy, one double osteotomy). A perforator-supported EAOM island was used in seven cases to reconstruct the inferior surface of the tongue [Figure 2]. In one patient with SCC of the retromolar area, an EAOM island was used to close the middle throat defect and the skin island was used to close the maxillary tuberosity defect [Figure 3]. Reconstruction was successfully performed in seven out of eight patients (overall success rate 88%). In one case, partial muscle necrosis was observed in the IAOM and EAOM flap due to venous thrombosis. The necrotic parts of the muscles were excised, and the wound healed by secondary intention. Pulmonary thromboembolism, which occurred in one patient, was successfully treated with low-molecular-weight heparin injections. Seven of the eight patients were decannulated and resumed an oral diet postoperatively. In one patient, due to partial muscle necrosis and early local recurrence, the tracheostomy was maintained and enteral nutrition via a percutaneous endoscopic gastrostomy (PEG) tube was required. Speech intelligibility was good in six of the eight patients. No postoperative complications, such as hernia, were observed. All patients underwent adjuvant radiotherapy, chemotherapy, or chemoradiotherapy.

## 4. Discussion

The DCIA free flap with internal oblique muscle is a well-known method for reconstruction in cases of oral cavity neoplasms. Because the IAOM can be insufficient for extensive, multidimensional defects after the removal of an advanced carcinoma of the tongue, floor of the mouth, or gingiva, the additional preparation of a perforator-supported EAOM flap can be useful.

In mandibular reconstruction, the choice of using a particular microsurgical flap or its modification, or the use of multiple flaps depends on the surgeon’s experience, the localization and size of the bone and soft tissue defect, and the potential morbidity of the donor site [2].

Advantages of DCIA flaps include the large amount of soft tissue and bone volume, along with a favorable shape, height, and width for mandibular reconstruction, which make this flap a good choice for further dental restoration [1]. On the other hand, myo-osseous or osteomyocutaneous variants of the DCIA flap have well-known limitations such as muscle bulkiness for intraoral lining and tight attachment of the muscle or skin to the bone, which limits its mobility and deteriorates proper orientation of the flap in complex oromandibular defects [1,3,9]. Crevecoeur et al. described the tripartite microvascular flap composed of external, internal, and transverse muscles but without the bony part, which allowed reconstruction of the large defect of the tongue, floor of the mouth, and cheek [10]. Kai-xiong Li et al. described a DCIA myofascial iliac crest flap variant with bony fragment and abdominal external oblique fascia (6 × 8 cm in size) for combined reconstruction in oral-mucosal-mandibular defects in Brown’s Class I, II, and III [11]. Wu et al. presented a composite flap with part of the internal oblique, external oblique, and muscle fascia tissue (maximum 5 × 6 cm in size) [12]. This modification improves the thickness of the soft tissue for intraoral lining; however, tethering of the fascia to the bone impairs the mobility of the flap. For this reason, some authors use additional microsurgical tissue flaps (ALT, RFFF, lateral arm flap) for compound intraoral defects [3,9]. However, the use of additional flaps may exacerbate surgical challenges, lengthen operation time, and cause increased complications with potential donor site morbidities [9].

Another solution is harvesting the DCIA perforator flap (DCIAPF), which improves soft tissue mobility, reduces bulk, increases pedicle length, and enables better tissue adjustment in oromandibular defects [1]. Since being first described by Taylor et al. [4], the classic DCIA flap has undergone many modifications in recent years [1,2,3,9,13,14,15,16]. The most common is the osteocutaneous variant, composed of the iliac crest with skin island supported on a musculocutaneous perforator evolving from the deep circumflex iliac vessel branches [1,2,3,9,14,15,16]. Some authors use a superficial iliac perforator flap (SCIP); however, according to the literature, only 13% of superficial circumflex iliac arteries have a common trunk with the deep circumflex iliac artery, so this modification often requires additional anastomoses [2,16,17]. The osteomyocutaneous variant of the DCIAP flap with iliac crest, internal oblique muscle, and perforator-supported skin island was presented by Wechselberger et al. They managed to achieve a high mobility of the skin paddle along the vascular pedicle and osteomuscular part to cover complex tissue loss of the upper jaw, midface, and orbit [13].

There have been many publications regarding musculocutaneous perforators and their anatomical variations. Bergeron et al. found DCIA perforators in 92% of cases and presented their anatomy. The transverse branch of the DCIA, which lies along the iliac crest between the transversus abdominis muscle and the internal oblique muscle, sends one or two musculocutaneous perforators to the overlying skin before its main trunk anastomoses with the lumbar or iliolumbar arteries. Perforators were located on the superior aspect of the iliac crest, 5 to 10.5 cm posterior to the ASIS. According to Bergeron, the DCIA perforator should be located with a Doppler probe in a 4 × 6 cm rectangular area on the superior aspect of the iliac crest, 5 cm posterior to the ASIS [18]. Safak et al. found a series of musculocutaneous perforators in 70% of dissections and a single dominant perforator in 30% of dissections [3]. Similarly, other authors found dominant musculocutaneous perforators in 70% of cases and they were located mainly 1–3 cm above the iliac crest and 3–6 cm posterior to the ASIS [3,4,9]. Zheng et al. divided the course of the deep iliac circumflex artery into three segments—the inguinal segment, the medial-to-crest segment, and the superior-to-crest segment—with three types of DCIA perforators leaving from corresponding segments: the abdominal muscular branches, the iliac osteomuscular branches, and the terminal musculocutaneous perforator. They described a DCIA perforator flap with skin flap modification based on the terminal musculocutaneous perforator of the DCIA [19].

An innovative application of a perforator-supported EAOM island was described for monitoring the blood supply to the DCIA flap. The authors harvested the EAOM island based on the ascending branch of the DCIA. Nutrient vessels of the EAOM in around 94% originate from the DCIA branches [7]. Schlenz et al. divided the blood supply to the EAOM into four types: Type 1 (81.6%)—muscular branches derive from the DCIA 6–8 cm lateral to the ASIS; Type 2 (10.5%)—muscular branch originates from the DCIA at the height of the ASIS; Type 3 (2.6%)—muscular branch derives from the ascending branch supplying the internal oblique abdominal muscle; Type 4 (5.3%)—muscular branch derives from the iliolumbar artery [20].

In our study, all perforators were localized 1–3 cm above the iliac crest and 3–6 cm posterior to the ASIS, and can be classified as Types 1 and 2 EAOM blood supply according to Schlenz [20]. In one case, we were able to harvest a composite DCIA flap with IAOM attached to the crest and additionally, two perforator-based islands, EAOM and cutaneous, which were necessary for wider reconstruction [Figure 3]. We harvested both IAOMs attached to the crest and perforator-supported EAOM island.

A disadvantage of preparing an EAOM island based on the ascending branch, as presented by Zhang et al., is that it requires cutting through the IAOM, meaning that harvesting the IAOM would be impossible [7]. Additionally, according to Schlenz, the blood supply from the ascending branch of the DCIA involves only 2.6% of cases [20]. On the other hand, it allows lengthening of the pedicle of the EAOM island so that it could be placed in the submental or submandibular region for buried flap monitoring [7].

Some authors have already used muscle fascia tissue to cover mucosal defects, mostly in the anterior part of the mandible, with very good results. However, these were not perforator-supported flaps and were not mobile enough to reconstruct three-dimensional defects. Additionally, they were used as an alternative to the IAOM and were harvested instead of this muscle [11,12]. The maximum size of the myofascial flap harvested by Wu et al. [12] was up to 5 × 6 cm, which is in line with our study and EAOM flap sizes. In other studies, some authors have successfully elevated fascia flaps up to 6 × 8 cm in diameter [11]. It appears that the smaller dimension of the flaps reduces the risk of donor site morbidity. In all cases presented in our study, a synthetic mesh was placed between the transversus abdominis muscle layers and the external oblique layers and attached to the bone and margins of the internal oblique muscle. Moreover, primary suturing of the EAOM was performed without tension, which was easier due to iliac bone loss after flap harvesting. No postoperative complications, such as hernia, were observed.

When discussing the issue of complex oromandibular reconstruction, chimeric flaps based on the fibula and the anterolateral thigh region should be mentioned [21,22]. There are many modifications of the chimeric fibular flap, which may consist of bone, muscle, and multiple skin paddles. Its use seems particularly beneficial in through-and-through orofacial defects that require both outer coverage and inner lining [21]. The anterolateral thigh osteomyocutaneous free flap (ALTO) can also be used in the reconstruction of large bony and soft tissue defects. A disadvantage of this technique is the increased risk of femoral fractures [22].

Our study has certain limitations, namely that the sample size is very limited and the study was conducted at a single center.

## 5. Conclusions

Along with continuous development of implantology, the DCIA flap is currently becoming another standard therapy for mandibular replacement. The DCIA free flap with IAOM and perforator-supported EAOM flap is a reliable method for compound soft tissue and bone defects in maxillofacial reconstruction. The use of internal and external oblique abdominal muscles can be helpful in cases of three-dimensional soft tissue defects of the lower gingiva, floor of the mouth, or tongue, where the lower gingiva and floor of the mouth can be reconstructed using the IAOM, while the more mobile part of the tongue can be reconstructed with a perforator-supported EAOM island. The use of the perforator-supported EAOM flap allows for the closure of an additional defect without the need to harvest another microsurgical flap, which shortens the procedure time and limits complications at potential donor sites.

## Figures and Tables

**Table 1:** Patient characteristics.

Case	Age/Sex ^1^	Tumor Pathology ^2^	Tumor Stage (pTNM)	Tumor Localization	Adjuvant Treatment ^3^	Complications	Follow-Up (Months)
1	69/M	SCC	pT3N0M0	Floor of the mouth	RT	-	14
2	59/K	SCC	pT4aN0M0	Floor of the mouth	CHX	partial necrosis	34
3	39/M	SCC	pT4aN3bM0	Floor of the mouth	CRT		20
4	60/M	SCC	pT3N2cM0	Floor of the mouth	CRT		14
5	57/K	SCC	pT3N2bM0	Lower gingiva	CRT	-	12
6	73/K	SCC	pT4aN0M0	Lower gingiva	RT	Pulmonary thromboembolism	14
7	64/K	SCC	pT3N3bM0	Retromolar area	CRT		9
8	60/M	SCC	pT2N0M0	Retromolar area	CRT		4

^1^ F—female; M—male; ^2^ SCC—squamous cell carcinoma; ^3^ RT—radiotherapy; CRT—chemoradiotherapy; CTX—chemotherapy.

## Data Availability

The data presented in this study is available on request from the corresponding author.
